# Pelvic floor electromyography and urine flow patterns in children with vesicoureteral reflux and lower urinary tract symptoms

**DOI:** 10.1590/S1677-5538.IBJU.2018.0401

**Published:** 2018

**Authors:** Lida Sharifi-Rad, Seyedeh-Sanam Ladi-Seyedian, Hossein Amirzargar, Abdol-Mohammad Kajbafzadeh

**Affiliations:** 1 Pediatric Urology and Regenerative Medicine Research Center, Children's Medical Center, Pediatric Center of Excellence, Tehran University of Medical Sciences, Tehran, Iran; 2 Department of Physical Therapy, Children's Medical Center, Pediatric Center of Excellence, Tehran University of Medical Sciences, Tehran, Iran

**Keywords:** Vesico-Ureteral Reflux, Lower Urinary Tract Symptoms, Urinary Bladder

## Abstract

**Objective:**

To determine the different urine flow patterns and active pelvic floor electromyography (EMG) during voiding in children with vesicoureteral reflux (VUR) as well as presenting the prevalence of lower urinary tract symptoms in these patients. Materials and Methods: We retrospectively reviewed the charts of children diagnosed with VUR after toilet training from Sep 2013 to Jan 2016. 225 anatomically and neurologically normal children were included. The reflux was diagnosed with voiding cystourethrography. The study was comprised an interview by means of a symptom questionnaire, a voiding diary, uroflowmetry with EMG and kidney and bladder ultrasounds. Urine flow patterns were classified as bell shape, staccato, interrupted, tower and plateau based on the current International Children's Continence Society guidelines.

**Results:**

Of 225 children with VUR (175 girls, 50 boys), underwent uroflowmetry + EMG, 151 (67.1%) had an abnormal urine flow pattern. An active pelvic floor EMG during voiding was confirmed in 113 (50.2%) children. The flow patterns were staccato in 76 (33.7 %), interrupted in 41 (18.2%), Plateau in 26 (11.5%), tower in 12 (5.3%) and a bell shape or normal pattern in 70 (31.5%). Urinary tract infection, enuresis and constipation respectively, were more frequent symptoms in these patients.

**Conclusions:**

Bladder/bowel dysfunction is common in patients with VUR that increases the risk of breakthrough urinary tract infections in children receiving antibiotic prophylaxis and reduces the success rate for endoscopic injection therapy. Therefore investigation of voiding dysfunction with primary assessment tools can be used prior to treating VUR.

## INTRODUCTION

The association between vesicoureteral reflux (VUR) and lower urinary tract (LUT) dysfunction especially in older children who present with urinary tract infection (UTI) after toilet training, is well known. The incidence of LUT dysfunction in children with VUR was reported from 18% to 75% (1). In addition, VUR is diagnosed in one third of UTI patients (2). The overactive bladder (OAB) and dysfunctional voiding (DV) have been described in conjunction with VUR (1). Increasing of intravesical bladder pressure in these conditions can be responsible for the development of VUR in affected patients. It is notable that spontaneous resolution of VUR after treatment of bladder dysfunction has been observed in most of the patients who were diagnosed with VUR after toilet training (2).

It has been found in the recent studies that some children with VUR have been diagnosed with abnormal urodynamic findings including OAB during filling phase and the increased activity of external urethral sphincter during voiding phase (1). Additionally, it has been reported that spontaneous resolution of VUR and cure rate following endoscopic treatment in children with LUT dysfunction and VUR are less than in children with VUR without LUT dysfunction (3-6). Therefore, an undiagnosed and untreated underlying LUT dysfunction can be a considerable cause of surgical failure (7). History taking and physical examination are the hallmark diagnostic tools for evaluation of LUT dysfunction in children. However repeated urine flow studies in conjunction with electromyography (EMG) can also help to confirm diagnosis of different LUT conditions in toilet trained children (8).

The aim of the present study was to evaluate the different urine flow patterns and active pelvic floor EMG during voiding in children who had VUR after toilet training as well as presenting the prevalence of LUT symptoms in these patients.

## MATERIALS AND METHODS

After institutional review board approval, we retrospectively reviewed the charts of all patients diagnosed and were treated with VUR after toilet training at our center from Sep 2013 to Jan 2016. A total of 225 anatomically and neurologically normal children were enrolled in the study. Children were selected from patients diagnosed with VUR who had been visited at outpatient pediatric urology clinic at Children's Medical Center, Tehran, Iran. The patients were between 5 to 11 years old. The diagnosis of VUR was confirmed through voiding cystourethrography (VCUG) and was graded according to the International Reflux Study in Children grading system (9). Children with neuropathic bladder, anatomic defects and a history of diagnosis of VUR at birth or early in life before toilet training were excluded from the study. Urine flow patterns, voided volume, maximum urine flow rate (Qmax), average urine flow rate (Qave), presence or absence of EMG activity during voiding phase, and post void residual (PVR) volume were evaluated. Moreover, prevalence of different LUT symptoms was determined.

### Evaluations

All participants underwent complete urological work - up as well as physical and neurological evaluations. The study evaluations included urine analysis and urine culture to assess UTI, a voiding diary, a dysfunctional voiding symptom score, uroflowmetry (UF) with EMG, VCUG, kidney and bladder ultrasounds to evaluate upper urinary tract, hydronephrosis, anomalies, bladder capacity and PVR. Patients who had febrile UTI or hydronephrosis on ultrasound underwent VCUG. Expected bladder capacity was measured with regard to International Children's Continence Society (ICCS) recommendation, using the formula mL = [(age in years + 1) x 30] (8).

A filled out LUT symptom and bowel habit questionnaire was obtained from all participated patients / parents to assess any associated LUT symptom, bowel habits, and other associated clinical manifestations. For any included patients, we noted and recorded gender, age, abdominal / flank pain, a history of UTI with or without fever, daytime incontinence, enuresis, urgency, infrequent voiding and / or holding maneuver and bowel dysfunction (constipation alone, fecal soiling alone or constipation plus fecal soiling). Enuresis was defined as wetting the bed at least once a week (10). Constipation was defined with regard to Rome III criteria (11). At least two UF / EMG in separate sessions were performed on all patients in accordance with the ICCS recommendations (8). Any medication with potential influence on bladder function was discontinued a week prior to performing of UF / EMG. Moreover, bladder ultrasound was performed to assess bladder capacity, PVR volume, the appearance of bladder wall and bladder neck, dilatation of lower ureteral and assessment of rectum for presence of a large stool mass. A written informed consent was obtained from participants.

### Urine flow patterns definition

With respect to the ICCS uroflow classification, the uroflow patterns were categorized into bell - shaped, staccato, plateau, interrupted, and tower shaped curves. The ICCS defines an interrupted - shaped curve as discrete segments of urine flow, separated by segments with zero flow. A staccato - shaped curve is defined as irregular and fluctuating during voiding but the flow is continuous and never reaches zero during voiding. A plateau - shaped curve is defined as a flattened and low - amplitude prolonged flow. Tower - shaped curve is defined as a sudden, high - amplitude curve of short duration and normal shape curve is defined as continues bell - shaped curve.

### Uroflow / EMG

All UF / EMG studies were performed with the use of a flowmeter with a spinning disc transducer (Tempo, Medtronic Urology, Skovlunde, Denmark) with respect to the ICCS guidelines and on strong need to void. Children voided in their normal position; relaxed posture with upper legs in a horizontal position and use of feet support for smaller children. To record pelvic floor EMG activity during voiding, two integrated biosensor EMG electrodes were placed on the perineum at 3 and 9 o'clock positions. The machine had a high quality audio monitor to recognize motor recruitment activity from activity caused by electrical artifact, such as wire movement or wetting of the electrodes. Children underwent at least two UF / EMG studies in separate sessions. All UF / EMG were done in a calm and equal environment. For each child, data regarding Qmax, Qave, voided volume, flow time and voiding time, as well as EMG activity during voiding were recorded.

### Statistical analysis

The Statistical Package of Social Science software (version 18; SPSS, Inc., Chicago, IL) was used for statistical analysis. Categorical data were reported as frequencies and percentages. Continuous data were reported as range and mean ± standard deviation (SD). To analyze data, chi - square or student t - test was executed. A P value of < 0.05 was considered statistically significant.

## RESULTS

### Study population

Among 463 patients with VUR who were assessed for eligibility, 238 patients with neuropathic bladder, anatomic defects and a history of diagnosis of VUR at birth or early in life before toilet training were excluded; only 225 children met inclusion criteria and were enrolled in the study. The data of 175 girls and 50 boys (mean age: 7.1 ± 2 years, range: 5 to 11) diagnosed with VUR (128 unilateral cases, 102 bilateral cases) were analyzed.

### UF / EMG outcomes

Abnormal urine flow patterns were observed in 151 (67.1%) patients. The flow patterns were staccato in 76 (33.7%), interrupted in 41 (18.2%), plateau in 26 (11.5%), tower in 12 (5.3%) and a bell shape or normal pattern in 70 (31.5%) (Table-1).

**Table 1:** Characteristics and urine flow patterns of all patients.

	Patient n. (%)			Urine flow patterns		
	Total	Normal (%)	Staccato (%)	Interrupted (%)	Plateau (%)	Tower (%)
Age (yr), mean ± SD (range)	7.1± 2 (5-11)	6.8±1.8 (5-11)	7.3 ± 2.1 (5-11)	7.6±2 (5-11)	6.6±2 (5-11)	6.4 ± 1.7 (5-10)
Female (%)	175	54 (30.8%)	62 (35.4%)	34(19.4%)	14 (8%)	11(6.2%)
Male (%)	50	16 (32.6%)	14 (28.5%)	7 (14.2%)	12 (24.4%)	1(2%)
**Total**	**225**	**70 (31.5%)**	**76 (33.7%)**	**41(18.2%)**	**26 (11.5%)**	**12 (5.3%)**
Bilateral VUR§	102	28 (27.4%)	38 (37.2%)	20 (19.6%)	12 (11.7%)	4 (3.9%)
Unilateral VUR§	128	43 (33.5%)	40 (31.2%)	22 (17.1%)	15 (11.7%)	8(6.2%)
EMG¥ activity during voiding	113	0 (0%)	69 (61%)	23 (20.3%)	15 (13.2%)	6(5.3%)

§ = vesicoureteral reflux;

¥ = electromyography

An active pelvic floor EMG during voiding was confirmed in 113 (50.2%) children. An active pelvic floor EMG during voiding was observed in 69 (61%) patients with a staccato urine flow pattern, 23 (20.3%) patients with an interrupted urine flow pattern, 15 (13.2%) patients with a plateau urine flow pattern and 6 (5.3%) patients with a tower curve. Overall 38 patients (25%) with a staccato, interrupted, tower and plateau urine flow patterns had a quiet pelvic floor EMG during voiding (Table-1).

Mean Qmax and mean Qave were 18.6 ± 8.5 (range: 6.3 to 45) and 9.2 ± 4.3 (range: 2 to 23) mL / sec, respectively. Mean voided volume was 203 ± 109 mL (range: 70 to 620) and mean PVR volume was 25.5 ± 22.8 mL (range: 0 to 105). Also, mean voiding time was 24.7 ± 14 sec (range: 6 to 76). Mean PVR volume was significantly higher in patients who had abnormal urine flow pattern in comparison with patients who had normal flow curve (32.3 ± 27.6 mL vs. 17.3 ± 5 mL, P < 0.04). There was no significant difference in UF measures between children who had abnormal urine flow pattern with and without positive EMG activity during voiding (Table-2). Of UF measures, mean voided volume in children with interrupted flow curve was significantly higher than in children with other curves (P < 0.05). Additionally, mean Qmax and mean Qave were significantly lower in children with plateau flow curve in comparison to children with normal curve (P < 0.05).

**Table 2:** Comparison of uroflowmetry measures and post-void residual volume between patients with normal and abnormal urine flow patterns.

Urine flow pattern	voided volume (mL)	Maximum urine flow rate (mL/sec)	Average urine flow rate (mL/sec)	Voiding time (s)	Residual volume (mL)
Bell shape	190±113	20.6 ± 7.6	10.9 ± 4.2	23.5 ± 14	17.3 ± 5
Staccato	194±117	20.1 ± 10.3	8.4 ± 4.1	21.7 ± 9.3	29.8 ± 24.8
P*	0.581	0.839	0.054	0.623	0.167
Bell shape	190±113	20.6 ± 7.6	10.9 ± 4.2	23.5 ± 14	17.3 ± 5
Interrupted	265 ± 87	15.1 ± 6.4	8.3 ± 4.4	27.7 ± 18.6	45 ± 44
P*	0.540	0.014	0.082	0.455	0.332
Bell shape	190±113	20.6 ± 7.6	10.9 ± 4.2	23.5 ± 14	17.3 ± 5
Plateau	209 ± 74	12.4 ± 5.3	6.5 ± 3.6	28.6 ± 14.3	30.8 ± 13.6
P*	0.351	0.01	0.02	0.450	0.299
Bell shape	190±113	20.6 ± 7.6	10.9 ± 4.2	23.5 ± 14	17.3 ± 5
Tower	133 ± 51	34.9 ± 6.6	14.6 ± 5.3	16.9 ± 6.4	9.2 ± 4
P*	0.053	0.034	0.014	0.04	0.04

Mean±SD;

* Student t test

Among different grades of VUR, grades II and III were more frequent in ureters of patients with and without abnormal urine flow pattern (Table-3).

**Table 3:** Lower urinary tract symptoms in different flow groups of patients.

	Patient n. (%)			Urine flow patterns		
	Total	Bell shape	Staccato	Interrupted	Plateau	Tower
Urinary tract infection n.%	201	67(34.8%)	72(35.8%)	32(16.6%)	21(10.9%)	9(4.4%)
Constipation n.%	56	20(35.7%)	18 (32.1%)	9(16%)	5(8.9%)	4(7.1%)
Daytime incontinence n.%	35	2(5.7%)	19(54.2%)	6(17.1%)	3(8.5%)	5(14.2%)
Nocturnal enuresis n.%	66	19(28.7%)	25(37.8%)	9(13.6%)	7(10.6%)	6(9%)
Urgency n.%	34	3(6.8 %)	12(35.2%)	4(11.7%)	3(6.8%)	12(35.2%)
Holding maneuver n.%	56	2(3.5%)	29(51.7%)	19(21.4%)	6(10.7%)	0

We reanalyzed the data of two different age groups (5 – 7 versus 8 – 11 years of age). There were 144 patients (64%, 26 boys and 118 girls) in the age range of 5 to 7 years old and 81 patients (36%, 23 boys and 58 girls) in the age range of 8 to 11 years old. There is no significant difference between the two age groups according to normal and abnormal urine flow patterns and EMG activity during voiding.

### Prevalence of lower urinary tract symptoms

Urinary tract infection, enuresis and constipation, respectively were more frequent features in patients with VUR. Of 225 children, 201 (89.3%) had a UTI history of whom 187 (93%) had at least 1 febrile UTI history. Constipation and enuresis were reported in 56 (24.8%) and 66 (29.3%) patients, respectively. In addition, 34 (15.1%) patients had urgency and 35 (15.5%) patients had daytime incontinence. Details of prevalence of LUT symptoms in VUR patients who had different urine flow patterns are shown in Table-4.

**Table 4:** Grade of VUR in different urine flow patterns.

	VUR grade I (unit)	VUR grade II (unit)	VUR grade III (unit)	VUR grade IV (unit)	VUR grade V (unit)
Bell shape	4	35	52	10	1
Staccato	5	49	46	17	3
Interrupted	4	21	27	6	3
Plateau	0	12	21	3	0
Tower	0	5	12	3	2

## DISCUSSION

There is a known association between VUR and LUT dysfunction in older children after toilet training. However, the exact relationship and natural history between VUR and LUT dysfunction are controversial. Prior studies have reported that children with LUT dysfunction and VUR have a higher frequency of UTIs (1). Moreover, they have a lower cure rate following endoscopic treatment (1, 12). One of the primary causes of surgical failure which prevents resolution of VUR in affected patients is an untreated underlying LUT condition (12). With respect to the association between LUT dysfunction and VUR, some authors have suggested that all children with VUR should be evaluated for abnormal urodynamic findings (7, 13).

UF with / without EMG is frequently used as a simple and non - invasive method to assess LUT function in pediatric urology practice. The appearance of the urine flow pattern accompanied with clinical neuro - urological evaluations, voiding diary and assessment of PVR through bladder ultrasound, can often provide enough information to decide the diagnosis and management (12). Mostly prior studies have used invasive urodynamic evaluation to assess patients with VUR. This study was undertaken to evaluate urine flow curves and measures in children who had VUR after toilet training by non - invasive UF / EMG. Additionally, the prevalence of LUT symptoms in these patients was determined. The results showed an abnormal urine flow pattern in 67.1% of patients. More than 50% of patients had a positive EMG activity during voiding. A staccato urine flow curve was seen in 33.7% of patients and also 18.2% of patients had interrupted urine flow curve. Only, 31.5% of patients had a normal urine flow curve. In addition, UTI, enuresis and constipation respectively, were more frequent manifestations in patients with VUR.

As regard to abnormal urine flow pattern, staccato and interrupted curves were more frequent in our patients. According to the ICCS guidelines, staccato urine flow pattern presents DV and interrupted pattern shows underactive bladder (8). Each condition involves urinary stasis and can be associated with high storage volume. Functional bladder outlet obstruction in children with LUT dysfunction can cause voiding difficulty and storage symptoms as a consequence of bladder changes secondary to obstruction (8). Accordingly, intravesical pressure rises resulting in production and persistence of VUR (14). Ural et al. compared the clinical, demographic, urodynamic and prognostic characteristics related to VUR in 348 patients with idiopathic LUT dysfunction or DV. They reported that median maximum filling pressure was higher in the refluxing group compared to the non - refluxing group (40.0 vs. 34.0 cm H2O). Moreover, increased intravesical pressure could be the primary factor for inducing reflux in idiopathic LUT dysfunction (15).

UTI commonly occurs in children with VUR. The close association between VUR and UTI, especially febrile UTI, has been discussed in the literature (16). The results of the present study reveal that nearly 90% of our patients had a history of UTI. This finding is the most frequent feature in patients with normal and abnormal urine flow curve. Although 31.5% of our patients had normal urine flow pattern, most of these patients complained of at least a LUT symptom such as constipation and / or enuresis. On the other hand, several studies have shown correlation between VUR and bladder bowel dysfunction (BBD) (17). VUR and constipation are well - recognized co - morbidities in children with bladder dysfunction. Treating constipation on the other hand has been shown to resolve urinary symptoms such as daytime wetting, enuresis and UTI up to 90% (18). In a survey by Koff et al. the influence of functional bladder and bowel disorder on the natural history of patients with primary reflux was assessed (19). The authors evaluated 143 pediatric patients who had either spontaneous resolution of VUR or had breakthrough infection which led to surgical management. They reported the rate of BBD was higher in children with breakthrough infections (77% vs. 23%). Moreover, they concluded that BBD adversely affected the results of reimplantation and was present as a risk for recurrent UTI after resolving reflux (19).

Additionally, the less cure rate of endoscopic treatment of VUR in children with BBD in comparison with children without BBD has been reported (4, 17). The results of mentioned studies strongly support that urodynamic evaluation can provide valuable information regarding the diagnosis, treatment and prognosis of children with VUR. Concomitant treatment for bladder dysfunction including pharmacotherapy, standard urotherapy (hydration, diet, timed voiding and toilet training) and biofeedback that can effectively help to spontaneously cure VUR or to achieve optimal results of anti - reflux surgery (12, 20, 21). Van Batavia et al. described their experience using dextranomer / hyaluronic acid copolymer in the patients whose VUR persisted despite targeted therapy for their LUT condition. They reported that the highest resolution rates of VUR were seen in patients with either DV or detrusor underutilization or in patients who had least symptoms prior to injection (3).

The main limitations of this study were small sample size and single center study. In addition, this was not a prospective study and we did not include a control group. A prospective study with a larger sample size is needed to offset these limitations.

## CONCLUSIONS

The results of present study have shown that two third of patients who had VUR after toilet training present an abnormal urine flow pattern. Additionally, more than 50% of the patients had an active pelvic floor EMG during voiding. Although one third of patients had normal urine flow pattern, most of mentioned patients complained of at least a LUT symptom. Therefore, evaluation and treatment of LUT dysfunction should be an essential part of the initial assessment and management of a child with VUR. Furthermore, treatment of LUT dysfunction would be supported to postpone definite surgical correction in these patients in order to improve surgical outcome.

## ABBREVIATIONS

BBD: = bladder bowel dysfunction
DV: = dysfunctional voiding
EMG: = electromyography
ICCS: = International Children's Continence Society
LUT: = lower urinary tract
OAB: = overactive bladder
Qave: = average urine flow rate
Qmax: = maximum urine flow rate
VCUG: = voiding cystourethrolgraphy
VUR: = vesicoureteral reflux
UTI: = urinary tract infection
